# Rheumatoid Vasculitis: Is It Always a Late Manifestation of Rheumatoid Arthritis?

**DOI:** 10.7759/cureus.5790

**Published:** 2019-09-28

**Authors:** Muhammad M Anwar, Ezza Fatima Tariq, Usman Khan, Muhammad Zaheer, Sardar H Ijaz

**Affiliations:** 1 Biochemistry, King Edward Medical University (KEMU) / Mayo Hospital, Lahore, PAK; 2 Nephrology, Oklahoma University Health Sciences Center, Oklahoma City, USA; 3 Internal Medicine and Nephrology, University of Oklahoma Health Sciences Center, Oklahoma City, USA; 4 Internal Medicine, Unity Health System, Rochester, USA; 5 Internal Medicine, University of Oklahoma Health Sciences Center, Oklahoma City, USA

**Keywords:** vasculitis, rheumatoid arthritis

## Abstract

Rheumatoid vasculitis (RV) is an infrequent complication of longstanding severe rheumatoid arthritis (RA). The active vasculitis associated with rheumatoid disease occurs in about 1%-5% of the patient population. RV is a manifestation of “extra-articular” rheumatoid arthritis and involves the small- and medium-sized arteries in the body. Newer RA treatments, including biologic therapies, offer a broader array of potential therapeutic options, although no controlled trials exist to guide treatment. In general, following tissue confirmation of the diagnosis, the severity of organ involvement and disease manifestations can guide treatment decisions. We want to alert clinicians of this unique yet severe complication of RA which has high morbidity and mortality. We describe a thought-provoking case of a 44-year-old male with past medical history (PMH) of hypertension who presented with over three-month history of lower extremity (LE) swelling, discoloration, and ulceration. Arthralgias with constitutional symptoms (fatigue, weight loss), large pericardial effusion, was found to have leukocytoclastic vasculitis along with rheumatoid factor (RF) >650, and anti-cyclic citrullinated peptide (anti-CCP) antibodies >300, low C4 and normal C3. Pericardial fluid appeared serous, exudative, showed histiocytes, multinucleated giant cells and necrotic debris consistent with rheumatoid effusion. Skin, right shin, punch biopsy showed epidermal necrosis from underlying occlusive vasculopathy. Skin, left lower back, punch biopsy showed focal leukocytoclastic vasculitis. The patient was started on high dose steroids with marked improvement in the symptoms, Rituximab was planned awaiting QuantiFERON to be negative. Pan-CT angiography of the whole body was negative for any vascular changes ruling out polyarteritis nodosa (PAN).

## Introduction

Rheumatoid vasculitis (RV) is an uncommon presentation of a common disease, i.e., rheumatoid arthritis (RA). It is the most serious extra-articular complication of rheumatoid arthritis and can cause high rates of morbidity and mortality. Traditionally, it affects a subset of patients with established disease. Virtually any organ system can be involved with the skin and peripheral nervous system involved most commonly [1]. As there are no specific signs and symptoms, the diagnosis relies on the exclusion of other causes of vasculitic involvement and on the histopathological demonstration of necrotizing vasculitis.

## Case presentation

A 44-year-old male with past medical history of hypertension presented to us with over three months history of lower extremity (LE) swelling, discoloration, and ulceration. The patient also had weakness, arthralgias, particularly hands which resulted in the limiting activities of daily living, constitutional symptoms (fatigue, weight loss), and bilateral lower extremity fluid collections.

**Figure 1:**
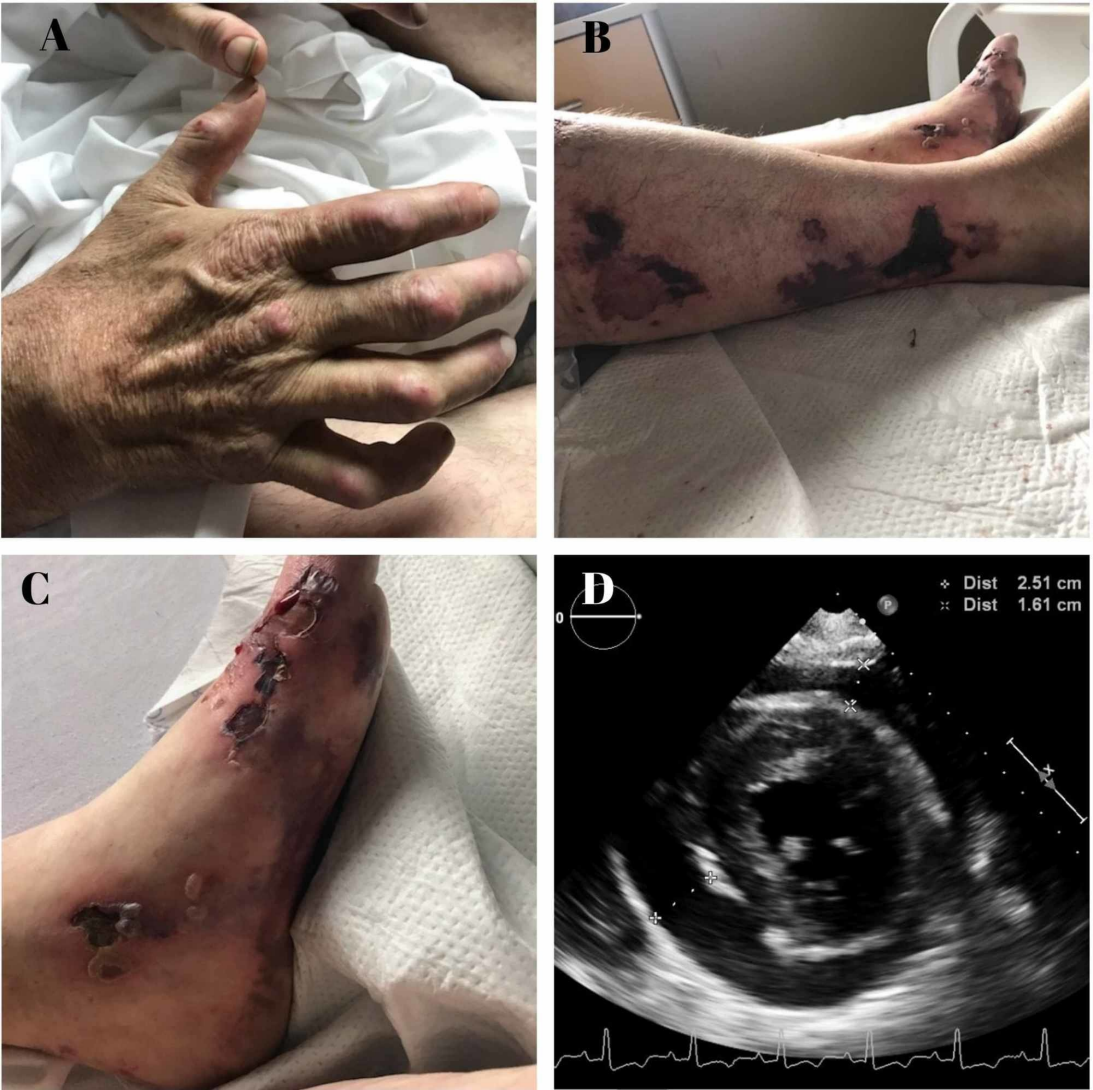
(A) Rheumatoid arthritis involving hands. (B & C) Skin manifestations of rheumatoid vasculitis. (D) Transthoracic echocardiogram showing large pericardial effusion.

Musculoskeletal examination revealed bilateral wrists, metacarpophalangeal and proximal interphlangeal joint swellings, flexion contractures of the hands bilaterally, lumbrical muscle wasting (Figure 1A). Multiple ulcerative non-purulent lesions with eschars were found on multiple areas on the skin (Figure 1B, 1C). Examination of other organ systems did not reveal any abnormality.

CT thorax/abdomen/pelvis showed moderate to large pericardial effusion with some mild pericardial enhancement, concerning for pericarditis, without any evidence of tamponade. Moreover, multiple rim-enhancing fluid collections, concerning for abscesses, were demonstrated in the bilateral lower extremities on CT angiography: largest was located between the gastrocnemius and soleus on the left with numerous tiny fluid collections extending the course and appeared to be communicating with other small collections; another along the posterior margins of the proximal right tibial metaphysis, collection along the superficial margin of the right and left gastrocnemius muscles.

Serological titers revealed negative anti-nuclear antibody (ANA), negative anti-neutrophil cytoplasmic antibodies (ANCA) with positive proteinase 3 (PR3) (unclear significance) RF > 650, and anti-CCP antibodies >300, low C4, normal C3. The pericardial fluid was drained (Figure 1D) and was found to be serous and exudative, and showed histiocytes, multinucleated giant cells and necrotic debris consistent with rheumatoid effusion.

Punch biopsy of the skin from right shin showed epidermal necrosis from underlying occlusive vasculopathy, whereas, punch biopsy of the skin from left lower back showed focal leukocytoclastic vasculitis. PAN was ruled out based on CT angiography of the whole body, which was negative for any vascular changes, thus ruling out PAN.

The patient was started on high dose steroids with marked improvement in the symptoms. Rituximab was initiated after QuantiFERON was found to be negative. He was meticulously tracked and treated in Rheumatology clinic at one- and two-month post discharge. He received two doses of rituximab, and high dose prednisone for a month. Steroids were tapered off after the 1st follow-up visit; methotrexate therapy was initiated on weekly basis, gradually titrated to reach a maximum of 30 mg per week. The patient reported a marked improvement in his symptoms after the initiation of methotrexate with disappearance of morning stiff and reduction in pain. On examination, complete subsiding of hand swelling, reduction in hand stiffness and only minimally present lower limb edema were noted.

## Discussion

Rheumatoid arthritis is a multisystem disease with recognized varied extra-articular presentations from the classic arthritic picture of the disease. Rheumatoid vasculitis is a serious but fortunately rare presentation of this disease involving small and medium blood vessels, reported to have involved skin, peripheral nerves, central nervous system and internal viscera with associated peripheral neuropathy (often motor), digital gangrene, nail bed infarcts and palpable purpura [1]. Typically, the patient has long-standing RA with severe joint deformities from underlying arthritis as well as high titers of immunological markers [2].

Even though there are no universal criteria for RV, diagnosis can usually be made on a combination of patient medical history, physical examination, specific laboratory tests, and histopathological examination (with evidence of necrotizing vasculitis involving small to medium-sized vessels, showing fibrinoid necrosis and mononuclear and neutrophil infiltration of the vessel walls) of any of the areas involved [3]. Rheumatoid vasculitis may sometimes be confused with polyarteritis nodosa (PAN) as both involve small- and medium-sized arteries by immune complex deposition, however, PAN shows areas of skipped lesions (the beads on a string presentation). Scott and Bacon criteria for the diagnosis of RV include the following: the presence of one or more of the following in a patient with RA: (i) mononeuritis multiplex or peripheral neuropathy; (ii) peripheral gangrene; (iii) biopsy evidence of acute necrotizing arteritis plus systemic illness (e.g., fever, weight loss); (iv) deep cutaneous ulcers or extra-articular disease (e.g., pleurisy, pericarditis, scleritis); if associated with typical digital infarcts or biopsy evidence of vasculitis. Other causes of such lesions, such as atherosclerosis and diabetes mellitus, should be excluded [4].

Multiple risk factors for RV have been reported that include long-standing RA, male, smoking, rheumatoid nodules and HLA class I and class II genotypes [5]. Overall, the incidence of rheumatoid vasculitis has declined over the past 30 years from 7.9 to 3.9 per million, which is attributed to advances in immunosuppression therapy and more effective treatment strategies of RA [6].

RV presented early in the course of RA in our patient is contrary to the widely held belief that RV is a late complication of RA. In fact, a few other clinicians have also reported the early onset of RV [7-9] and one case in which diagnosis of RV predated the diagnosis of RA [10]. In our patient, the diagnosis of RV is concurrent with the diagnosis of RA. Features that favor a diagnosis of RV at presentation even in the absence of polyarthritis are high serum RF titer and low serum C4 levels.

Traditionally, corticosteroids and cyclophosphamide have been used for the treatment of rheumatoid vasculitis. With the advent of biologically active agents, the paradigm of RV has been shifted to the employment of anti-TNF agents and Rituximab [11, 12].

With a short time period from the development of symptoms to diagnosis of this disease and methodical ruling out of pertinent differential diagnoses, we hope a better prognosis and early recovery of the patient highlighting the critical relationship between the onset of symptoms and institution of a treatment regime.

## Conclusions

A high degree of suspicion is required for prompt recognition and treatment in order to secure a better prognosis and avoid complications and morbidity associated with this severe form of rheumatoid vasculitis. Moreover, as in other forms of vasculitides, the definitive diagnosis can only be made histopathologically.
